# Characteristics and potential functional effects of long insertions in Asian butternuts

**DOI:** 10.1186/s12864-022-08961-3

**Published:** 2022-10-28

**Authors:** Yidan Chen, Yating Miao, Weining Bai, Kui Lin, Erli Pang

**Affiliations:** MOE Key Laboratory for Biodiversity Science and Ecological Engineering and Beijing Key Laboratory of Gene Resource and Molecular Development, College of Life Sciences, Beijing Normal University, Beijing, 100875 China

**Keywords:** Asian butternut, Pangenome, Long insertion, Combination, Functional effects

## Abstract

**Background:**

Structural variants (SVs) play important roles in adaptation evolution and species diversification. Especially, in plants, many phenotypes of response to the environment were found to be associated with SVs. Despite the prevalence and significance of SVs, long insertions remain poorly detected and studied in all but model species.

**Results:**

We used whole-genome resequencing of paired reads from 80 Asian butternuts to detect long insertions and further analyse their characteristics and potential functional effects. By combining of mapping-based and de novo assembly-based methods, we obtained a multiple related species pangenome representing higher taxonomic groups. We obtained 89,312 distinct contigs totaling 147,773,999 base pair (bp) of new sequences, of which 347 were putative long insertions placed in the reference genome. Most of the putative long insertions appeared in multiple species; in contrast, only 62 putative long insertions appeared in one species, which may be involved in the response to the environment. 65 putative long insertions fell into 61 distinct protein-coding genes involved in plant development, and 105 putative long insertions fell into upstream of 106 distinct protein-coding genes involved in cellular respiration. 3,367 genes were annotated in 2,606 contigs. We propose PLAINS (https://github.com/CMB-BNU/PLAINS.git), a streamlined, comprehensive pipeline for the prediction and analysis of long insertions using whole-genome resequencing.

**Conclusions:**

Our study lays down an important foundation for further whole-genome long insertion studies, allowing the investigation of their effects by experiments.

**Supplementary Information:**

The online version contains supplementary material available at 10.1186/s12864-022-08961-3.

## Background

Structural variants (SVs), which are defined as sequence variants at least 50 bp in size, can be classified as deletions, duplications, insertions, inversions, and translocations [1]. Compared with single-nucleotide polymorphisms (SNPs) and short insertions and deletions (Indels), SVs commonly affect a larger proportion of the genome; thus, SVs can have large phenotypic effects [2, 3]. Meanwhile, SVs are ubiquitous in plant genomes, and play important roles in adaptation and speciation [4, 5]. Especially, long insertions may harbor important functional sequences such as protein-coding or regulatory sequences [6], which underline their importance.

Recent advances in sequencing technologies and computational methodologies have provided incredible resolution to study SVs. Whole-genome resequencing has become an important source for identification of SVs [7] in addition to the efficient identification of SNPs [8]. The following three strategies are used to detect SVs: de novo assembly-based approaches, short-read alignment-based approaches, and long-read alignment-based approaches [9]. Accordingly, many methods have been proposed, such as Assemblytics [10], DELLY [11], and Sniffles [12]. Although de novo assembly-based approaches can detect insertions longer than 3,000 bp [13], the lack of haplotype representation is one major challenge [9] though there are methods such as chromosome-scale haplotype reconstruction [14] to account for this. Besides, de novo assembly requires high-depth sequencing, so the cost is high. Long-read mapping-based methods often perform better than short-read methods. However, currently, short-read-based methods are still widely used due to the accuracy and cost of the next-generation sequencing.

Due to the development of technologies and methodologies, SVs are increasingly being recognized as an important type of variation. In particular, studies investigating SVs in plants are increasing, and many studies show that SVs play important roles in adaptation, diversification, domestication and breeding [15]. Hamala et al*.* used 31 wild-collected cacao genomes to identify SVs and found that some individual SVs bear signatures of local adaptation, while some SVs may impair gene function by influencing gene expression [16]. Zhou et al. identified SVs from clonally propagated grapevine cultivars and their outcrossing wild progenitors [17]. These authors found that strong purifying selection acts against SVs, while some independent large, complex inversions have driven convergent phenotypic evolution. Kou et al.inferred SVs from whole-genome assemblies and long-read data [18]. These authors used SVs to study domestication and found that SVs contribute to the cost of domestication in rice. Guan et al.generated a high-quality de novo genome assembly of a flat-fruit peach cultivar, and identified a large inversion (1.67 Mb) that segregates perfectly with a flat-fruit shape [19]. Fuentes et al.identified regions of high SV frequency enriched in stress response genes, and demonstrated how SVs may help in finding causative variants in genome-wide association analyses by identifying SVs in 3,000 rice genomes [20].

Asian butternut (*Juglans* section *Cardiocaryon*) include the following species: *Juglans ailantifolia*, *J. mandshurica* and *J. cathayensis* [21]. As Asian butternuts have important economic and ecological value, many studies investigating Asian butternuts have been performed. Bai et al*.* assembled scaffold-level *J. mandshurica* genome [22], and then, the genome was updated to a chromosome-scale. Recently, a whole-genome resequencing of 80 Asian butternuts was conducted by Xu et al*.*, who highlighted the critical role of sex-biased dispersal in causing discordance between the nuclear and plastid genomes [23]. However, to date, no study investigating SVs in Asian butternuts has been reported. Therefore, the SVs in Asian white walnuts remain unknown. To address this gap, we used whole-genome resequencing data of 80 Asian butternuts to detect a type of SV, namely, long insertions. To decipher the effects of the putative long insertions, we further analysed the characteristics and potential functional effects of putative long insertions. Identifying long insertions by short paired-end sequencing data is a major challenge as many long insertions are often ignored by single short-read approaches [24]. Therefore, we further propose PLAINS (https://github.com/CMB-BNU/PLAINS.git), a pipeline for the discovery of insertions longer than 1,000 bp, by combining short read mapping-based and de novo assembly-based methods, and integrating the downstream analyses, including the structural annotation of genes and functional effects.

## Results

### Novel sequences in the pangenome of Asian white walnuts

**Fig. 1:**
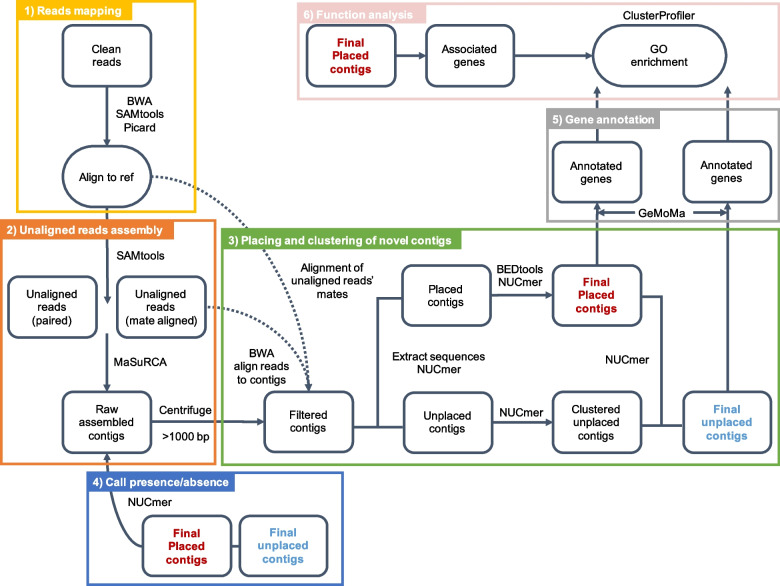
Pipeline for the prediction and analysis of novel sequences. The pipeline includes six parts: reads mapping, unaligned reads assembling, novel sequences placing, presence/absence calling, gene structure annotating, and function analysis

To obtain novel sequences in 80 Asian butternuts (Fig. S1), following the pipeline (Fig. 1), 600,739,223 reads unaligned to the *J. mandshurica* reference genome were extracted from 80 Asian butternuts (Table S1). MaSuRCA [25] was used to assemble the unaligned reads of each individual. We obtained 351,672 contigs with lengths greater than 1,000 bp (Table S2). Then, after removing the contaminated contigs, 109,556 contigs remained (Table S2). Furthermore, after filtering redundant contigs, we obtained 148 Mb of novel DNA distributed across 89,312 nonredundant contigs. Meanwhile, 1,439,531 bp occupying 0.97% of the novel DNA sequences were repetitive sequences, in which 1,411,288 bp occupying 0.96% of the novel sequences were transposable elements (TEs), including 1,400 bp SINEs, 188,066 bp LINEs, 715,989 bp LTRs, 124,481 bp DNA transposons, 44,257 bp rolling-circles, 121 bp penelopes, and 336,974 bp unclassified TEs. These repetitive sequences were dispersed in 6,245 novel contigs, in which 201 contigs were covered by TEs with coverage of more than 70%.

**Fig. 2:**
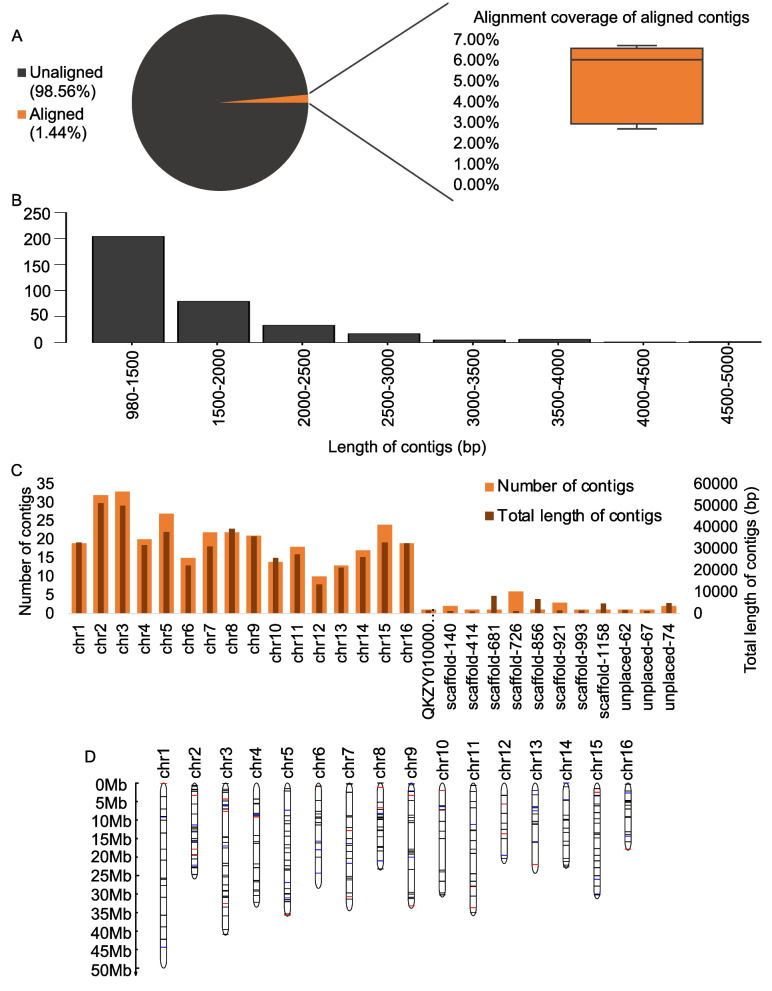
Overview of the placed contigs. **A**. Alignment of the placed contigs with the reference genome. **B.** Length distribution of the placed contigs. **C**. Total length and number of the placed contigs among 16 chromosomes and other scaffolds. **D**. Distribution of placed contigs among 16 chromosomes. Each line represents a placed contig, a red line indicates that the contig inserts into a gene, and a blue line indicates that the contig inserts into 5 kb upstream of a gene

Of these 89,312 contigs, 347 could be placed in the reference genome by one end, two-end placed contigs were not found, and neither two ends of 88,965 contigs could be placed in the reference genome (Table 1). To investigate the novel sequences, we realigned these contigs to the *J. mandshurica* reference genome by NUCmer in MUMmer v3.23 [26]. Of the 347 placed contigs, 1.44% of them could be aligned to the reference genome, and their maximum coverage was 6.67% (Fig. 2A); of 88,965 unplaced contigs, only one contig was aligned to the reference genome with 79.09% identity and 9.02% coverage. These findings suggest that the contigs were novel compared with the *J. mandshurica* reference genome.

**Table 1:** Novel sequences in the pangenome of Asian butternuts

	#Contigs	Total length (bp)	Mean length (bp)	Longest contig (bp)
Placed contigs	347	546,379	1,575	4,869
Unplaced contigs	88,965	147,227,620	1,655	73,342
Total	89,312	147,773,999	1,655	73,342

We further analysed the assembled novel contigs. The length distribution of the novel contigs is shown in Fig. 2B; the longest placed sequence was 4,869 bp, and the longest unplaced sequence was 73,342 bp (Table 1). We discovered 546,379 bp of the novel placed contigs with an average length of 1,575 bp, and 147,227,620 bp of unplaced contigs with an average length of 1,655 bp (Table 1). Regarding the 347 placed contigs, the placement locations were dispersed across the genome; of these placed contigs, 326 were located in chromosomes in the reference genome, and 21 were placed in the scaffolds. We also observed that chr2 contained the longest sequences, accounting for 51,203 bp, and chr12 contained the shortest sequences, accounting for 13,414 bp (Fig. 2C-D).

We searched for the existence of the novel sequences in the *J. cathayensis* reference genome to determine how many detected sequences were shared between *J. cathayensis* and *J. mandshurica* reference genomes. After additionally aligning our 347 placed contigs and 88,965 unplaced contigs to the *J. cathayensis* reference genome by NUCmer, we found that 159 placed contigs accounting for 246,217 bp and 222 unplaced contigs accounting for 346,828 bp were aligned to the *J. cathayensis* reference genome with ≥ 90% identity and ≥ 80% coverage. This finding implies that some novel sequences surely occur in *J. cathayensis* reference genome.

### Performance of PLAINS pipeline

We used simulated data to validate the PLAINS pipeline. First, 33 simulated DNA sequences were randomly inserted into the chr3 chromosome as the ground truth. After running the PLAINS pipeline, interestingly, 33 novel sequences happened to be assembled. Then, NUCmer was used to align the novel assembled contigs to the simulated sequences as the ground truth. We found that 33 assembled contigs were all aligned to the ground truth data with ~ 98.11% coverage, meaning that the precision and recall were both 100% for the assembling of novel sequences. We observed that 5 of 33 novel sequences could be located in consistent positions with the ground truth, while the other 28 novel sequences were all classified as unplaced. Because PLAINS pipeline adopted strict positioning strategies. If a sequence satisfies the following two conditions, it is considered to be located in the genome. First, “Region unambiguous” proposed by Sherman et al*.* [27] was used, i.e., > 95% of the aligned reads aligned to a region of a chromosome within 2 kb. However, 13 contigs could not match “Region unambiguous” at both of their ends. Second, a contig end must have a consistent exact match of at least 15 bases with the reference genome, while 15 contigs could not match the condition.

### Potential effects of the novel sequences

The novel sequences located in gene regions may disrupt the structure of genes, and further influence the function of genes. We observed that 61 genes were targeted by the putative long insertions. Although no significant GO terms were enriched for the 61 genes, genes involved in DNA repair (GO:0006281), flower development (GO:0009908) and glycolytic processes (GO:0006096) were observed, showing that these putative insertions may influence DNA repair, plant development, and glycolytic metabolism.

**Fig. 3:**
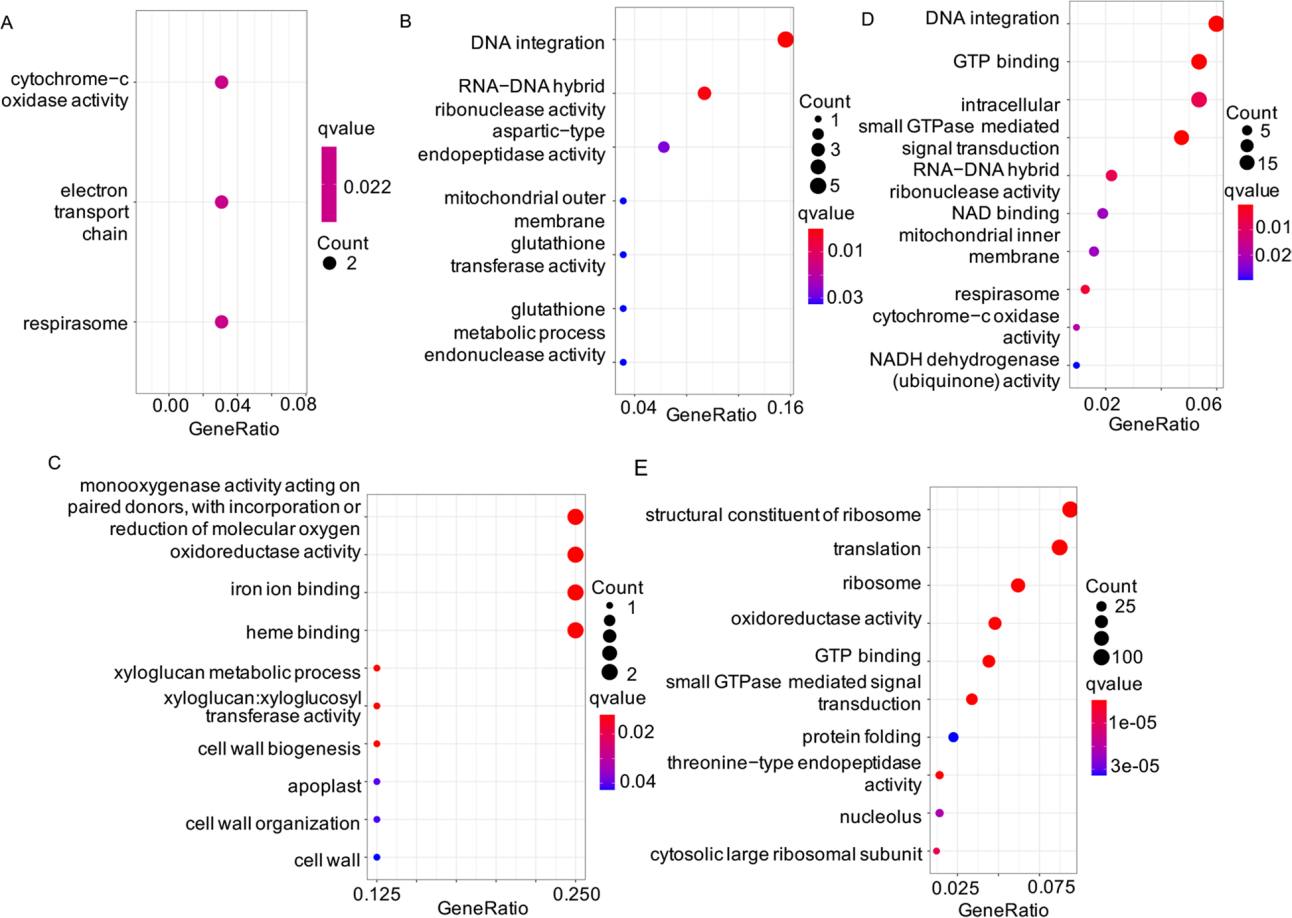
GO enrichment of the genes affected by the novel sequences. **A**. Genes whose 5 kb upstream were disrupted. **B**. Genes annotated by GeMoMa in 347 placed contigs harbored TEs. **C**. Genes annotated by GeMoMa in 347 placed contigs without TEs. **D**. Genes annotated by GeMoMa in 88,965 unplaced contigs harbored TEs. **E**. Genes annotated by GeMoMa in 88,965 unplaced contigs without TEs

The novel sequences located in upstream regions may affect the expression of corresponding genes. Therefore, we extracted genes whose 5 kb upstream sequences were targeted by the long insertions. We obtained 106 genes that were significantly enriched in cytochrome − c oxidase activity (GO:0004129), electron transport chain (GO:0022900) and respirasome (GO:0070469) (Fig. 3A), suggesting that the putative insertions may damage the regulation of genes related to cellular respiration.

The novel sequences might include genes. To understand the function of the novel sequences, GeMoMa [28] was used to annotate these contigs. Of the 347 placed contigs, 96 were annotated by 111 genes, with an average length of 697 bp and an average of 2 exons. For the 111 genes in the placed contigs, 101 genes in the contigs harbored TEs were significantly enriched in DNA integration (GO:0015074) (Fig. 3B), and the other 10 genes in contigs without TEs were significantly enriched in monooxygenase activity (GO:0004497) and oxidoreductase activity, acting on paired donors, with incorporation or reduction of molecular oxygen (GO:0016705) (Fig. 3C). Of the 88,965 unplaced novel contigs, 2,510 were annotated by 3,256 genes. Among the annotated genes in unplaced novel contigs, 691 in contigs harbored TEs were also significantly enriched in DNA integration (GO:0015074) (Fig. 3D), and 2,565 genes in contigs without TEs were significantly enriched in GO terms related to translation, such as structural constituent of ribosome (GO:0003735) and translation (GO:0006412) (Fig. 3E), indicating that these putative insertions produced additional copies of genes related to translation.

### Novel putative insertions are highly low frequency

Regarding the above novel sequences, we further investigated whether they were present/absent in each Asian butternut sample. NUCmer was used to align all raw contigs from MaSuRCA of 80 individuals to our final placed and unplaced contigs. In total, 910 contigs were shared by at least two individuals (Table 2). Regarding the 88,965 unplaced contigs, each individual contained 1,174 of these novel sequences on average, implying that most of the unplaced contigs (88,349 occupying 99.3%) were singleton (Table 2). Regarding the placed contigs, each individual contained 83 of these novel sequences on average (Table 2). On average, each placed contig was shared by 19 individuals (Table 2). The placed contigs were insertion sequences not found in *J. mandshurica* reference genome and defined as putative long insertions. Here, we further analysed the placed contigs.

**Table 2:** Presence/absence of novel contigs

	#Contigs	Mean number of insertions per individual	Mean number individuals per insertion
Placed contigs	347	83 (23.92%)	19 (of 80)
Unplaced contigs	88,965	1174 (1.32%)	1 (of 80)
Total	89,312	1257 (1.41%)	1 (of 80)
Nonprivate only	910	152 (16.70%)	13 (of 80)

**Fig. 4:**
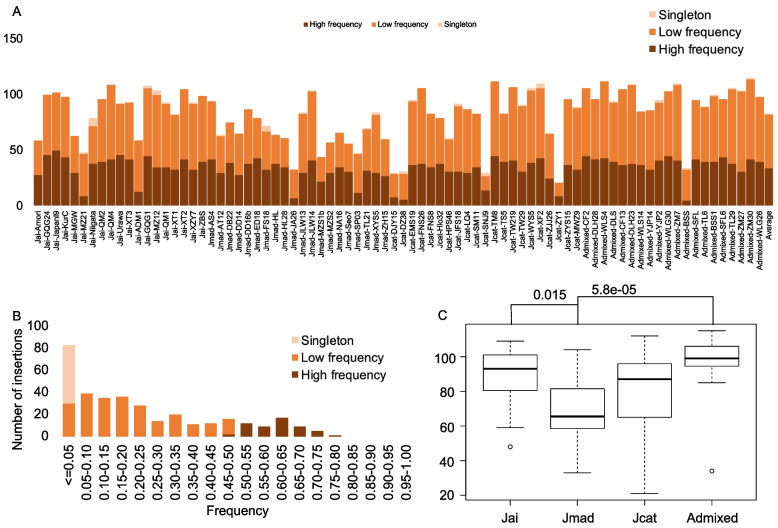
Statistics of present/absent sequences. **A**. Number of each insertion category in each individual. **B**. Frequency of 347 putative insertions in 80 individuals. **C**. Insertions numbers in each species, *P* values of pairwise Wilcox test are marked if they are less than 0.05. Jai: *Juglans ailantifolia*, Jmad: *J. mandshurica*, Jcat: *J. cathayensis*, Admixed: hybrid samples of *J. mandshurica* and *J. cathayensis*

We classified the placed contigs into the following four categories: singleton (identified in only one individual), low frequency (identified in more than one individual but less than 40 individuals), high frequency (identified in 40 or more individuals but less than 80 individuals), and fixed (identified in all individuals). For 347 putative long insertions, no fixed insertions were found, 55 putative insertions were identified as high frequency, 239 putative insertions were identified as low frequency, and 53 putative insertions were identified as singleton. At the individual level, most of putative insertions were low frequency, and the fewest putative insertions were singleton (Fig. 4A). On average, there were 34, 48 and 1 high frequency, low frequency and singleton putative insertions in each individual, respectively. The frequency of these putative insertions also showed that most of putative insertions (76%) were common in 80 individuals (frequency > 0.05), and only 24% of the putative long insertions were rare (frequency ≤ 0.05) (Fig. 4B).

At the species level, the median putative insertion numbers in *J. ailantifolia* individuals, *J. mandshurica* individuals, *J. cathayensis* individuals and admixed individuals were 93, 66, 87 and 98, respectively (Fig. 4C). Because we used the *J. mandshurica* genome as the reference genome, there were the fewest putative insertions in *J. mandshurica* individuals. Interestingly, we found that there were the most putative insertions in admixed individuals, significantly more than those in *J. mandshurica* individuals (Wilcoxon test, *p* = 5.8e-5, Fig. 4C).

### Unique putative long insertions in multiple species

**Fig. 5:**
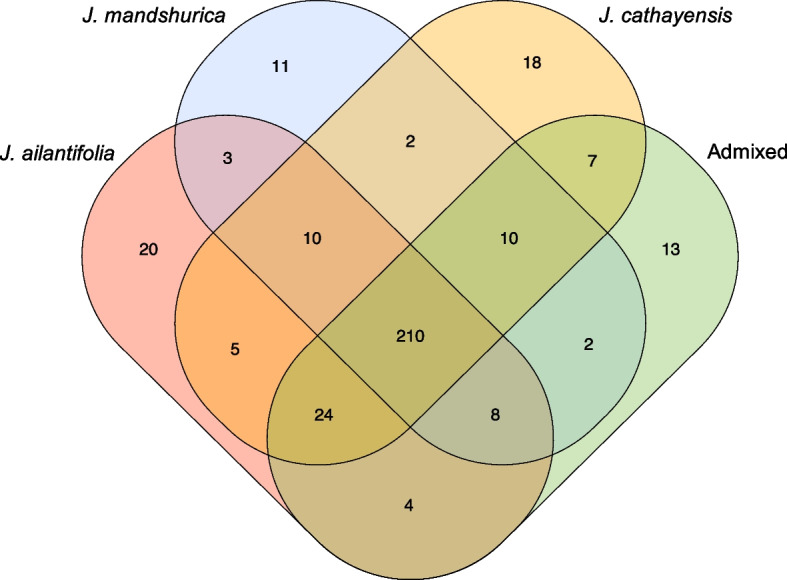
The Venn diagrams of putative long insertions

At the species level, we classified the putative long insertion into the following two categories: in a single species and in multiple species. Among the 347 putative long insertions, only 62 putative insertions were found in a single species (Fig. 5). These putative insertions were involved in the body of 12 genes, upstreams of 18 genes, and 13 genes annotated by GeMoMa (Table 3).

**Table 3:** Putative insertions in a single species

Species	Insertions	Disrupted genes	Disrupted upstream	Genes annotated by GeMoMa
*J. ailantifolia*	20	3	6	2
*J. mandshurica*	11	3	2	5
*J. cathayensis*	18	3	5	1
Admixed	13	3	5	5
Total	62	12	18	13

In *J. ailantifolia*, there were 20 unique putative long insertions involving in 3 genes disrupted (Table 3). Among the disrupted genes, *JMA024564.1* was annotated by DNA repair (GO:0006281), flower development (GO:0009908), positive regulation of cell division (GO:0051781) and regulation of transcription, DNA-dependent (GO:0006355) (Table S3). In *J. mandshurica*, there were 11 unique putative long insertions involving in 3 genes disrupted, and *JMA009133.1* was annotated by response to stress (GO:0006950) (Table S3). In *J. cathayensis*, there were 18 unique putative long insertions involving in 5 genes disrupted by upstream, and *JMA001495.1* was involved in lipid metabolic processes (GO:0006629) (Table S3). These findings show that the putative insertions may influence the development of butternuts in *J. ailantifolia*, the response to stress in *J. mandshurica*, and lipid metabolism by affecting the expression of corresponding genes in *J. cathayensis* individuals.

In the admixed individuals, there are 13 putative long insertions. The bodies of 3 genes and upstream of 5 genes were disrupted by putative insertions, and 5 genes were annotated by GeMoMa (Table 3). Among the disrupted genes, *JMA003816.1* was annotated by carbohydrate metabolism (GO:0005975) (Table S3), showing that it may influence metabolism. Regarding genes inserted upstream, *JMA027094.1* is involved in the respiratory chain (GO:0070469) (Table S3), and *JMA025266.1* is involved in chloroplast organization and biogenesis (GO:0009658) and riboflavin biosynthesis (GO:0009231) (Table S3), showing their potential influence on cell respiration and chloroplast organization. Among the 5 genes annotated by GeMoMa, one gene is paralogous of *JMA017750.1* involved in cell wall organization (GO:0071555) and xyloglucan metabolic process (GO:0010411) (Table S3), suggesting that the putative insertions could potentially influence cell wall biogenesis by increasing the copy number of *JMA017750.1*.

### Putative long insertions shared among all species

Meanwhile, we observed that most of the putative insertions existed in all species. Therefore, we investigated the long insertions shared among all species. Overall, 210 putative insertions of the 347 putative long insertions were shared among all species (Fig. 5). They were dispersed up to 16 samples in *J. ailantifolia* and *J. mandshurica* individuals and up to 19 samples in *J. cathayensis* and admixed individuals (Fig. S2). These putative insertions were involved in 41 disrupted genes, 69 genes disrupted by upstream, and 65 genes annotated by GeMoMa.

For 41 disrupted genes, though no significant go terms were detected, some genes involved in 'de novo' pyrimidine nucleobase biosynthetic process (GO:0006207), nucleotide-excision repair (GO:0006289), damaged DNA binding (GO:0003684), and DNA replication (GO:0006260) were observed, suggesting that these putative insertions may disrupt genes involved in DNA repair and replication. Genes whose 5 kb upstream were inserted by putative long insertions were involved in the cellulose biosynthetic process (GO:0030244), DNA repair (GO:0006281), response to auxin (GO:0009733) and response to stress (GO:0006950), showing that these putative insertions might affect the expression level of genes related to cellulose biosynthesis, plant development and stress response. Interestingly, 60 annotated genes in novel contigs harbored TEs significantly enriched in DNA integration (GO:0015074) and RNA–DNA hybrid ribonuclease activity (GO:0004523) (Fig. S3A). And 5 genes in novel contigs without TEs were significantly enriched in monooxygenase activity (GO:0004497) and oxidoreductase activity, acting on paired donors, with incorporation or reduction of molecular oxygen (GO:0016705) (Fig. S3B), which may suggest their roles in redox reaction.

## Discussion

Despite the prevalence and significant importance of SVs, long insertions remain poorly detected and studied in all but model species. The identification of long insertions requires long reads or high-coverage sequencing to assemble genomes [9], limiting their detection and further understanding. To address this problem, we proposed an integrated pipeline, PLAINS, which can identify long insertions without high-coverage sequencing reads by aligning reads to the reference genome and assembling unaligned reads. Additionally, we used the pipeline to identify and annotate putative insertions in Asian butternuts. Beyond the pangenome resources and putative long insertions we provide in Asian butternuts, our study also offers an example of how short reads can be used to construct a pangenome, detect putative long insertions, annotate the insertions, and understand their potential influence.

Pangenomics enables calling and genotyping/phasing SVs comprehensively [29]. To date, most pangenomes were constructed using a single species; here, we constructed a pangenome representing multiple related species (*J. ailantifolia*, *J. mandshurica*, *J. cathayensis* and admixed individuals) representing a higher taxonomic group [30]. In total, we discovered 546,379 bp of the novel placed sequences and 147,227,620 bp of unplaced sequences. We found a lower percentage of placed sequences in Asian butternuts than in humans [27]. On the one hand, due to their high repeatability, the assembly of plant genomes is presumably even more challenging than the assembly of vertebrate genomes [31]. Therefore, we speculate that the high repetitiveness in Asian butternuts renders the de novo assembly sequences shorter compared with those of vertebrates, implying more unplaced sequences than those in humans. On the other hand, the similarity of the sequences was low, leading to more novel sequences not being located in the reference genome, which enlightened us to use relatively loose strategies for locating.

Species-specific SVs affect the response to the environment where the species is living [32]. Although, Species-specific putative long insertions in Asian butternuts occurred in few individuals, they were associated with different functions. In *J. ailantifolia*, there were putative insertions associated with flower development and positive regulation of cell division. Flowering is critical for the reproduction of angiosperms [33], and cell division is key for adaptation to environmental conditions [34]. In *J. mandshurica*, there were putative insertions associated with the response to stress; as plants are continuously exposed to a broad range of environmental stresses [35], the stress response plays a key role in the environmental response. In *J. cathayensis*, there were putative insertions associated with lipid metabolic processes. Lipids are major components of biomembranes and play a role in the response to environmental stress, such as heat stress [36], and mutants of genes involved in lipid metabolism almost invariably result in changes in lipid composition in the cell [37]. In admixed individuals, there were putative insertions associated with the respiratory chain, chloroplast organization and cell wall biogenesis. The respiratory chain is a fascinating and highly optimized system for energy transduction [38]. The chloroplast has the additional function of sensing ever-changing environmental conditions [39] and is the central switch of the plant's response to cold and light stress [40], and cell walls can provide structural support during growth and protection from biotic and abiotic stresses [41]. Species-specific putative long insertions would be important for finding potential response to the microenvironment, when our pipeline will be applied in other plant species in the future.

Most (60.52%) of putative insertions we found were shared in all species (Fig. 5), and potentially have some basement functional effects. Our functional analysis suggests that these putative insertions were associated with DNA repair, DNA replication and response to auxin. DNA replication is central to cell proliferation [42], and auxin is an important plant hormone that can act as a signalling molecule to regulate a vast number of developmental responses [43] by regulating genes at the transcriptional level [44]. Meanwhile, DNA repair coupled with DNA replication maintain genome stability to ensure high fidelity and complete copying of the genome [45] and occur preferentially faster at transcriptionally active genes as it could couple with transcription to maintain active genome integrity [46]. Thus, these putative insertions may have positive effects on DNA replication and transcription and, thus, were shared in all species.

Although our study helps to deepen the understanding of the Asian butternut genome, several limitations should be mentioned. First, although we detected larger insertions by combining mapping and de novo assembly-based approaches, compared to methods without de novo assembly, heterozygous insertions were missed by the fact that assembly contigs only represent one haplotype. Therefore, we could only obtain the frequency of occurrence in the samples, instead of the allele frequency. Second, the annotation of the novel sequences was based on the proteins of the target species, indicating that we could only obtain the copies of existing genes and may have ignored many potentially functional genes, especially when the reference genome was poorly annotated. Third, although we observed some putative insertions were inserted in gene bodies and other putative insertions altered regulatory regions, further elucidation of the biochemical cases and consequences of insertions is needed to understand the evolution of Asian butternuts.

## Conclusions

In conclusion, we provided pangenome resources for Asian butternuts, including putative long insertions. Meanwhile, our study offers a case study of how short reads can be used to construct pangenome, detect long insertions, annotate the insertions, and understand their potential influence. Moreover, we launched an automated pipeline for identifying and annotating putative long insertions missing by SV identifying methods based on short-read alignment using whole-genome resequencing paired-reads.

## Methods

Our pipeline aims at to aid in the identification and functional analysis of the putative long insertions, including 1) mapping reads, 2) assembling unaligned reads, 3) locating novel sequences in the genome, 4) calling presence/absence, 5) annotating gene structure and function, and 6) function analysis. A schematic of the pipeline is shown in Fig. 1. It took 34 min to obtain unaligned reads from 2 samples (genome size about 520 Mb), 148 min to assemble reads, 25 min to filter and mask contigs, and 5 min to locate and cluster contigs, totaling 212 min.

### Data sources

Four types of data were used in our study, including genomic sequence data, genome structural annotation and genome functional annotation of *J. mandshurica* and whole-genome resequencing of paired-reads of 80 Asian butternuts. The genomic sequence and resequencing data were downloaded from NCBI under the BioProject accession number PRJNA356989, which were published in two articles [23, 47]. The genomic annotations were downloaded from the Juglans Genus Genome Projects (http://cmb.bnu.edu.cn/juglans). We used 150 bp paired-end reads of whole-genome resequencing data of 80 individuals with an average of 30X coverage per individual genome.

The leaf sample were collected from 80 adult individuals throughout the whole range of Asian butternuts in northern and southern China, the Korean Peninsula, Japan, and Taiwan Island. They included 19 *J. ailantifolia* samples, 20 *J. mandshurica* samples, 21 *J. cathayensis* samples, and 20 hybrid samples of *J. mandshurica* and *J. cathayensis* (admixed) [23] (Fig. S1).

### Read mapping and assembly

For each sample, the paired clean reads were aligned to the *J. mandshurica* reference genome using BWA v0.7.17 [48] with the parameter "-M". Then, SAMtools v1.11 [49] was used to convert the obtained SAM files to BAM files with the default parameters. Next, PCR duplicates were marked with MarkDuplicates in Picard Toolkit v2.25.2 (http://broadinstitute.github.io/picard/) with the default parameters.

We extracted unaligned reads from those BAM files by SAMtools and then assembled all unaligned reads with MaSuRCA v4.0.4 [25] software, respectively. If neither mate in a read pair aligned to the *J. mandshurica* reference genome, we treated them as paired reads with a fragment size of 300 bp, and if only one read in a pair was not aligned, we treated it as an unpaired read.

We retained only assembled contigs longer than 1000 bp and used Centrifuge v1.0.4 [50] to evaluate the contigs. Any contigs labelled by Centrifuge as non-chordates or humans were removed. Meanwhile, the remaining contigs were masked by RepeatMasker v4.1.2 (http://www.repeatmasker.org/RepeatMasker/) with the low-complexity option off (–nolow).

### Placing contigs in the *J. mandshurica* reference genome

We attempted to place the assembled contigs in a location on the *J. mandshurica* reference genome using paired reads. First, BWA was applied again to align the unaligned reads from the read pairs with one end read had aligned to the *J. mandshurica* reference genome to the assembled contigs. Then, for reads that were aligned within 500 bases of the end of a contig, the alignments of their paired reads to the reference genome were further examined to determine whether the contig had an unambiguous placement in the reference genome. Using the definition of “region unambiguous” proposed by Sherman et al*.* [27], i.e., > 95% of the aligned reads aligned to a region of a chromosome within 2 kb, contigs with at least one end having unambiguous placements were detected. For these contigs, the 200 bp sequence of each contig end and its corresponding sequences on the reference genome, including the unambiguous region and its 500 bp flanking regions, were extracted. Then, NUCmer in MUMmer v3.23 [26] was applied to align them. If a contig end had a consistent exact match of at least 15 bases with the reference genome, we considered it was exactly placed.

The placed contigs were obtained and further divided into the following three groups: two-end placed, left-end placed and right-end placed contigs. To cluster the contigs in each group, we used BEDtools v2.30.0 [51] with the parameter "-d 100 -c 4 -o distinct" to group all placed contigs located at approximately the same location, and the longest contig per group was selected as the representative contig. Because we did not find two-end placed contigs, we further clustered those one-end placed contigs based on NUCmer alignment. If these representative contigs were placed within 5 kb of one another on the reference genome and their alignments exhibited ≥ 98% identity and ≥ 95% coverage, we considered them belonging to one contig cluster, merged them and selected the longest contig in the merged cluster as the final sequences. Finally, the contigs were trimmed to remove sequences that aligned to the reference genome by NUCmer with the default parameters. Thus, we obtained a putative long insertion set that included all trimmed exactly placed contigs.

### Clustering and filtering of unplaced contigs

To remove the redundant unplaced contigs, first, we used NUCmer to align all unplaced contigs against one another with the parameter " –maxmatch –nosimplify -l 31 -c 100". For each alignment with an identity ≥ 98% and any coverage ≥ 95%, the longest contig remained. Then, we used NUCmer to align the remaining unplaced contigs to the untrimmed putative long insertion set and removed the contigs producing alignments with ≥ 90% identities and ≥ 80% coverages. Finally, we obtained the unplaced contigs.

### Evaluation of PLAINS pipeline

To validate PLAINS pipeline, we extracted the chr3 chromosome of the reference genome, which contained the maximum number of putative long insertions reaching 33. Then, we randomly produced 33 DNA sequences as the ground truth, of which length distribution was similar to that of the putative long insertions. Next, we performed VISOR v1.1.2 [52] to randomly inserted the 33 DNA sequences into the chr3 chromosome and generated a haplotype-resolved FASTA file. To simulate paired-end sequencing reads, ART v2.5.8 [53] was applied to obtain 30X paired-end sequencing reads from the haplotype-resolved FASTA file. PLAINS pipeline was performed to detect long insertions. NUCmer was used to compare the results identified by the PLAINS pipeline with the ground truth.

### Annotation of repetitive sequences

To annotate repetitive sequences in 89,312 novel sequences, RepeatModeler v.2.0.3 [54] was used for identification of de novo transposable element families. Then, RepeatMasker v4.1.2 was applied for repeat sequences annotation, while the de novo library, and repeat sequences of plants from RepBase (release 20,181,026) [55] and Dfam (version 3.6) [56] were used as the repetitive sequence database.

### Comparison with the *J. cathayensis* reference genome

To further verify the assembled contigs, we downloaded a *J. cathayensis* reference genome from a previous study [57] (http://xhhuanglab.cn/data/juglans.html). We aligned the putative long insertion set and the unplaced contig set to the *J. cathayensis* reference genome by NUCmer with the default parameters. The contigs producing alignments with ≥ 90% identities and ≥ 80% coverages were considered as consistent contigs with the *J. cathayensis* reference genome.

### Functional analysis of putative long insertions

To explore their effects, the putative long insertions were classified into the following three types: gene regions, regulator regions (5 kb upstream of a gene) [16] and intergenic regions. We extracted genes whose sequences or 5 kb upstream were targeted by long insertions. We further used GeMoMa software [28] to predict the genes in the insertion sequences by homology-based methods using genome functional annotation of the *J. mandshurica* genome. Annotated genes were further divided into two types: genes in contigs harbored TEs and genes in contigs without TEs. Moreover, the ClusterProfiler [58] package in R was used to perfume a GO enrichment analysis of each gene list.

### Calling the presence/absence of each individual

To call the presence/absence of novel sequences in 80 individuals, we used NUCmer to align all raw contigs from MaSuRCA of 80 individuals to our final putative insertion set and unplaced contig set with the default parameters. If any alignments were annotated by NUCmer as identical, we defined this insertion present in this individual; otherwise, we defined the insertion absent. Thus, we further divided our insertions into the following four groups: 1) in one sample (singleton), 2) in more than one sample but less than 40 samples (low frequency), 3) in 40 or more samples but less than 80 samples (high frequency), and 4) identified in 80 samples (fixed).

## Supplementary Information

**Additional file 1: Fig. S1:** Geographic origin and PCA of the 80 individual Asian butternuts. Fig. S2 Number of putative long insertions shared among all species in each species. Fig. S3 GO enrichment of the genes influenced by putative long insertions shared among all species. Description of data: Fig. S1 Geographic origin and PCA of the 80 individual Asian butternuts. Fig. S2 Number of putative long insertions shared among all species in each species. Jai: *Juglans ailantifolia*, Jmad: *J. mandshurica*, Jcat: *J. cathayensis*. Fig. S3 GO enrichment of the genes influenced by putative long insertions shared among all species. A. Genes annotated by GeMoMa in putative long insertions with TEs. B. Genes annotated by GeMoMa in putative long insertions without TEs.

**Additional file 2: Table S1:** Reads for each individual. Table S2 Assembled contigs. Table S3 Genes influenced by unique putative long insertions in each species.

## Data Availability

All data that support this study are included within the article and its additional files. The genomic sequence and resequencing data were downloaded from NCBI under the BioProject accession number PRJNA356989. The genomic annotations were downloaded from the Juglans Genus Genome Projects (http://cmb.bnu.edu.cn/juglans). Code used for identifying novel sequencing and analysing functional effects of putative long insertions are available at: https://github.com/CMB-BNU/PLAINS.git.
