# Supplementary material for: Characteristics and potential functional effects of long insertions in Asian butternuts
Source: BMC Genomics. 2022 Oct 28;23:732. doi: 10.1186/s12864-022-08961-3 (PMC9617325; doi:10.1186/s12864-022-08961-3)
Supplement: Supplementary file 1 — Additional file 1: Fig. S1: Geographic origin and PCA of the 80 individual Asian butternuts. Fig. S2 Number of putative long insertions shared among all species in each species. Fig. S3 GO enrichment of the genes influenced by putative long insertions shared among all species. Description of data: Fig. S1 Geographic origin and PCA of the 80 individual Asian butternuts. Fig. S2 Number of putative long insertions shared among all species in each species. Jai: Juglans ailantifolia, Jmad: J. mandshurica, Jcat: J. cathayensis. Fig. S3 GO enrichment of the genes influenced by putative long insertions shared among all species. A. Genes annotated by GeMoMa in putative long insertions with TEs. B. Genes annotated by GeMoMa in putative long insertions without TEs. [file 12864_2022_8961_MOESM1_ESM.docx]

**Fig. S1 Geographic origin and PCA of the 80 individual Asian butternuts [1]****.**

**Fig. S2 Number of putative long insertions** **shared among all species in each species.** Jai: *Juglans ailantifolia*, Jmad: *J. mandshurica*, Jcat: *J. cathayensis.* Each bar represents an insertion.

**Fig. S3 GO enrichment of the genes influenced by putative long insertions shared among all species. A.** Genes annotated by GeMoMa in putative long insertions with TEs. **B.** Genes annotated by GeMoMa in putative long insertions without TEs.

**Reference**

1. Xu LL, Yu RM, Lin XR, Zhang BW, Li N, Lin K et al. Different rates of pollen and seed gene flow cause branch-length and geographic cytonuclear discordance within Asian butternuts. New Phytol. 2021;232(1):388-403.
